# Urticaria and other mimickers of urticaria

**DOI:** 10.3389/falgy.2024.1522749

**Published:** 2025-02-13

**Authors:** María Isabel Rojo-Gutierrez, Carol-Vivian Moncayo-Coello, Alejandra Macias Weinmann, Rene Maximiliano Gomez, Luis Felipe Ensina, Ivan Cherrez-Ojeda, Pedro Piraino Sosa, Patricia Latour Staffeid, Marylin Valentin Rostan

**Affiliations:** ^1^ Hospital Juárez de México, Mexico City, Mexico; ^2^ Service of Allergy and Clinical Immunology of Hospital Juarez de México, Mexico City, Mexico; ^3^ Allergy and Clinical Immunology Service of Hospital Universitario Dr Eleuterio González, Monterey, Mexico; ^4^ Catholic University of Salta, Salta, Argentina; ^5^ Federal University of São Paulo, São Paulo, Brazil; ^6^ Institute for Allergology, Charité – Universitätsmedizin Berlin, Corporate Member of Freie Universität Berlin and Humboldt-Universität zu Berlin, Berlin, Germany; ^7^ Universidad Espiritu Santo Samborondon Ecuador, Ecuador, Ecuador; ^8^ Universidad Nacional Pedro Henríquez Ureña, Santo Domingo, Dominican Republic; ^9^ Prívate Service British Hospital Allergy & Immunology, Montevideo, Uruguay

**Keywords:** urticaria, mast cells, vasculitis, wheal, papule

## Abstract

Urticaria is a mast cell-dependent skin disease characterized by the presence of hives, angioedema, or both in the absence of systemic symptoms. It may be acute, or chronic. (1) Acute urticaria (AU) is common in children, affecting boys and girls equally. Chronic urticaria (CU) affects adult women more (3). AU affects more than 20% of the population and CU 0.1 and 1.5%. There are many pathologies that do not meet the clinical criteria for urticaria, despite being called urticarias, which leads to erroneous diagnoses and inconclusive epidemiology. This review attempts to clarify when we should consider urticaria as such and what are the diagnoses that can be considered urticaria without being so.

## Introduction

Urticaria is a disease characterized by the presence of hives, angioedema, or both, typically without systemic symptoms. It can be classified as acute, lasting less than 6 weeks, or chronic, persisting for more than 6 weeks (1). It is common across all ages groups, with acute forms affecting children more frequently, and, occurring equally in both boys and girls. On the other hand, chronic urticaria are common throughout the world, and Latin America having the highest incidence. This situation represents a significant burden on health, especially in chronic cases that occur more in adult life, with women being the most affected in a 2:1 ratio in relation to men (2, 3).

Urticaria is a condition frequently evaluated in an allergy consultation. However, the first assistance of patients usually involves emergency services, dermatology and even toxicology, with an expected difference in management due to the approach that each of them gives to the problem. The prevalence of acute urticaria is high, with 20% of the population having had it at some time. However chronic forms occur between 0.1% and 1.5% in Latin America and Asia (4).

The characteristic lesions are pruriginous hives or wheals (edema of the dermis) surrounded by erythema. Visible lesions are evanescent, as they disappear and appear without a trace unless the recurrent lesions harden or leave an abrasion due to scratching. When these lesions cause residual hyperpigmentation of the area, other conditions (e.g., vasculitis) must be taken into consideration (2).

**Figure 1:**
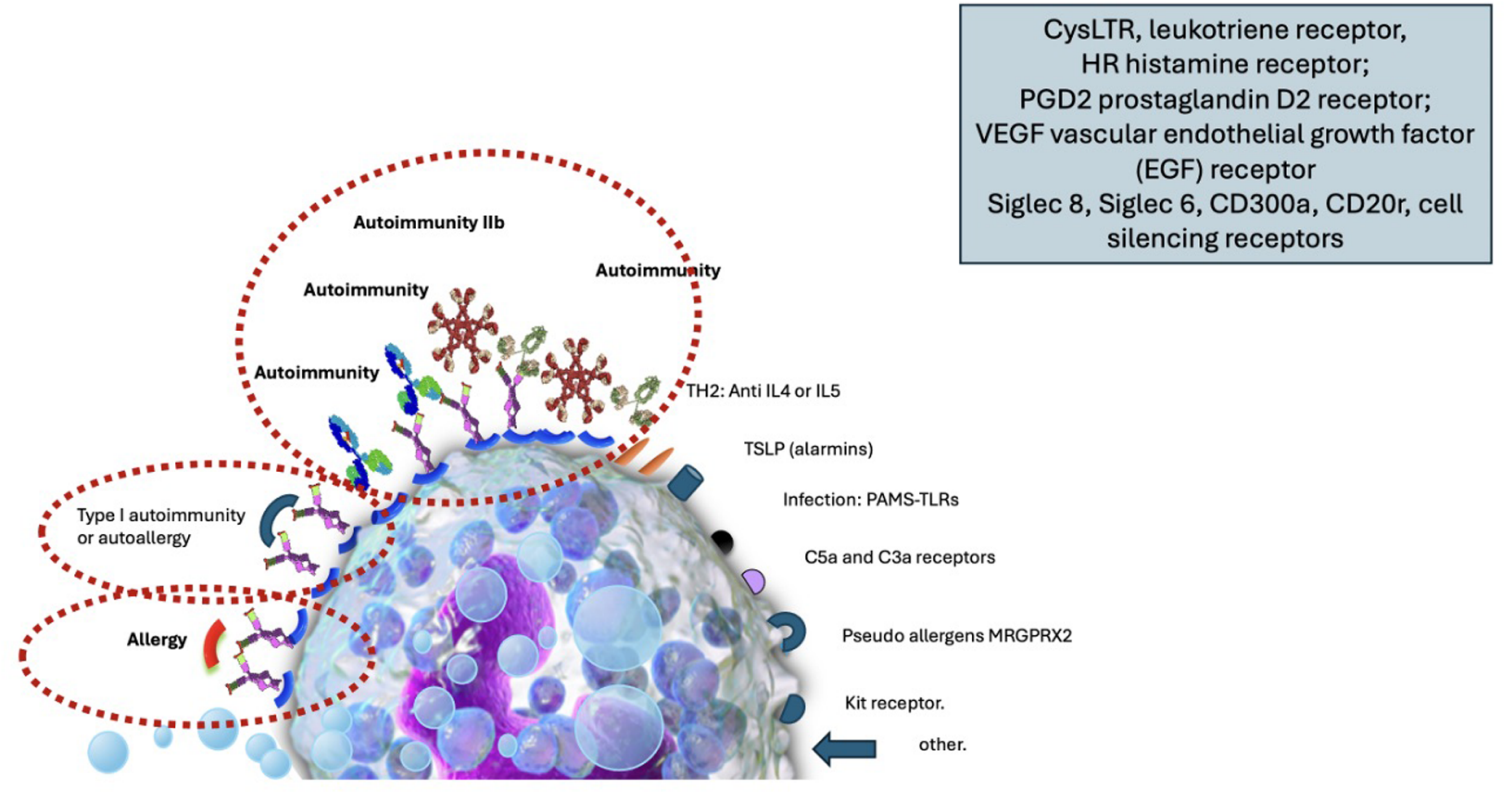
Image of the mast cell with its surface receptors capable of activating degranulation by various mechanisms of action: the allergic phenomenon, autoimmunity type I (autoallergy), autoimmunity IIb, receptors for TH2 interleukins (IL4, IL5), receptors for alarmins and thymic stromal lymphopoientin (TSLP), receptors for molecular patterns associated with pathogens recognized through toll-like receptors (TLR). Receptors for MRGPRX2 (Mas-related G-protein coupled receptor member), receptors for soluble factors of the C3 and C5 complex (anaphylatoxin), cKit receptor, colony-stimulating factor receptor, other receptors such as those for histamine, leukotrienes, prostaglandins, etc., including receptors capable of silencing the cell such as Siglec 8, Siglec 6, CD300a and CD200r.

The lesions can be subclassified by etiology into spontaneous or inducible. It is also possible to classify by the mechanism that produces it, demonstrating that there are multiple receptors on the surface of mast cells capable of activating or inhibiting their degranulation. (Figure 1 Degranulation mechanisms in urticaria.)

**Figure 2:**
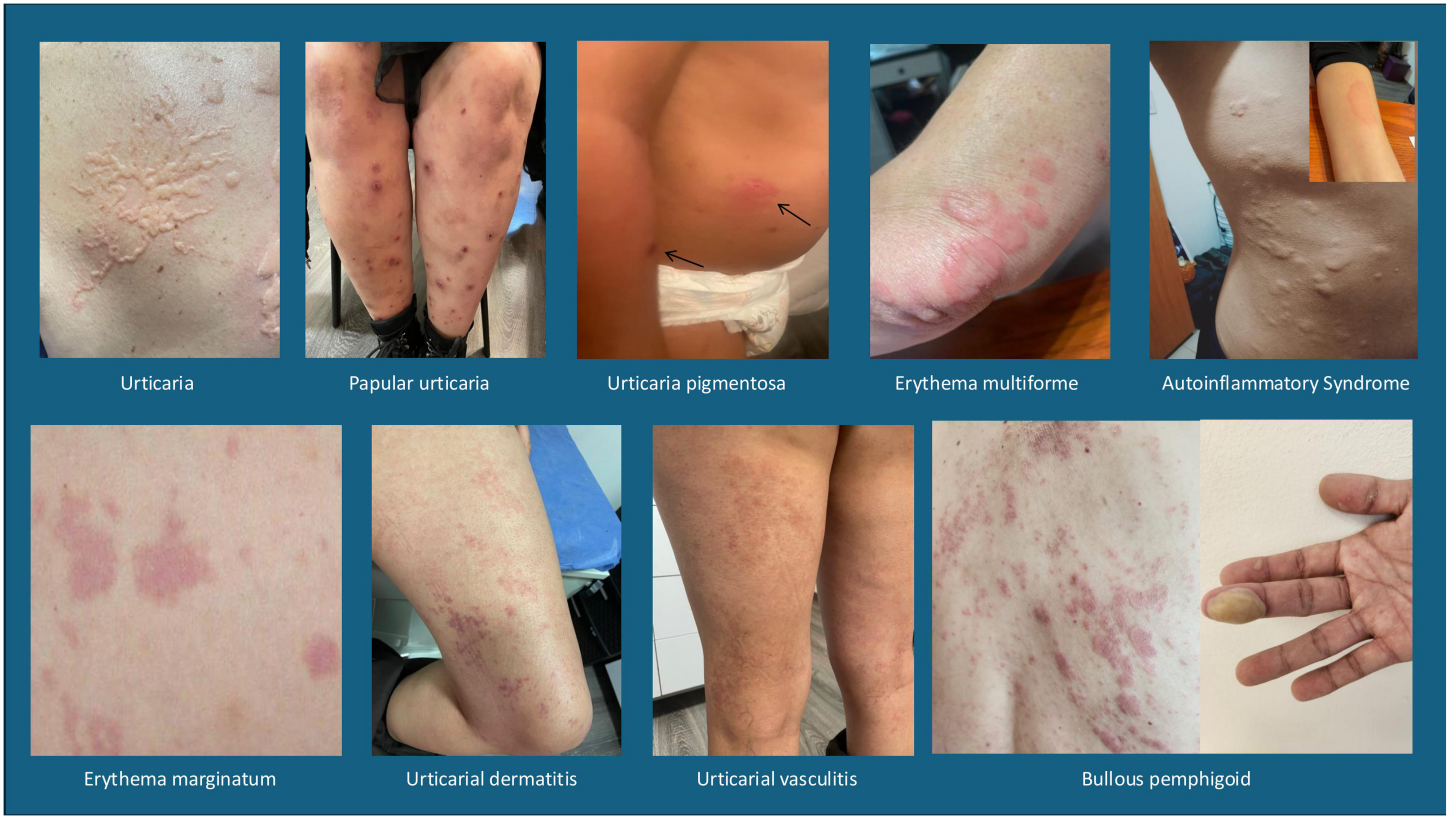
Images of differential diagnoses in urticaria: vasculitic urticaria, Urticaria pigmentosa or mast cell activation syndrome, papular urticaria, erythema multiforme, erythema marginatum, urticarial dermatitis, autoinflammatory syndrome and bullous pemphigus.

Unfortunately, there are many pathologies in the literature that do not meet the clinical criteria for urticaria but are included as such, causing erroneous diagnoses and inconclusive epidemiology, depending on the site where the analysis is performed. Some examples of these pathologies are: vasculitic urticaria, urticaria pigmentosa, papular urticaria, as well as other lesions that are often confused by morphology at some point such as erythema multiforme, erythema marginatum, urticarial dermatitis, mast cells disorders, autoinflammatory syndrome, bullous pemphigus, etc. In Figure 2 several images with lesions frequently related to the diagnosis of urticarial are shown, and for this particular report will be called imitators or mimickers (Figure 2).

The pathophysiological mechanisms associated with each of these mimickers are different, presenting signs of hypersensitivity or not. When this occurs, an immediate or delayed type might be involved, with activation of humoral immunity to produce immunoglobulins against foreign or self antigens, or a mechanism of cellular immunity in relation to late processes, with infiltration and damage produced by immune cells.

Urticaria can be also a manifestation of other pathologies such as infections(both viral including COVID 19,fungal, parasitic and bacterial), paraneoplastic syndrome, drugs reactions, anaphylaxis, etc. While the presence of characteristic lesions of urticaria at the onset, and the rest of the lesions can guide through differential diagnoses, a brief description is provided in Table 1.

**Table 1:** Characteristic lesions and differential diagnoses in urticaria.

Diagnosis	Hives (edema of the dermis)	Macule (erythema, vasodilation)	Papule (Small, soft bump on the skin without pus, without xerosis.)	Vesicle (fluid-filled sacs or bags)	Ulcer (lesion due to ischemic necrosis of the skin or subcutaneous tissue)	Petechia (very small dermal or subcutaneous hemorrhage)	Itching	Other
Urticaria	x						xxx	Edema of the dermis that occurs with or without angioedema (edema in subcutaneous cellular tissue)
Urticaria vasculítica,	x	x				x	+++/-	Inflammation of small vessels in the skin, causing red spots that may or may not be itchy, but it does cause burning and hyperpigmentation of the skin.
Urticaria pigmentosa,	x	x					-	It is a type of mastocytosis, it has reddish-brown lesions that can form hives or blisters when caressed. (Darier's sign)
Urticaria papular,			x	x			++/-	Papular urticaria is a papular vesicular reaction to insect and arachnid bites
Erythema multiforme	x	x	x	x	x		+/-	Erythematous ring rings with an external erythematous area and which may have a central blister that encloses an area of normal skin tone.
Erythema marginatum		x	x				-	Serpentine lesions with defined borders, rounded reddish spots, do not itch or pain, have a migratory pattern
Urticarial dermatitis		x		x	x/-		++/-	Bullseye-shaped lesions, reddish border, light or dark center
Autoinflammatory syndrome,	x						+	Recurrent episodes of fever, urticaria, inflammation in localized sites of the body, absence of autoantibodies and tendency to familial aggregation.
Pemphigus		x	x	x	x		-	Large, fluid-filled blisters.

The pathophysiological mechanisms associated with each of these mimetics are different, with or without hypersensitivity data, activating humoral immunity to produce antibodies and cellular immunity with the activation of multiple cells of the immune system including the epithelia.

Below we briefly describe the most important characteristics of each of the diagnoses that can be considered mimetic or at least that are frequently confused.

### Vasculitic urticaria (VU)

Vasculitic urticaria is a condition frequently associated with autoimmune phenomena with the presence of complex deposits in small cutaneous vessels with biopsies reporting cellular infiltration.

Vasculitic urticaria (VU) is a rare condition characterized by small vessel vasculitis with recurrent episodes of skin lesions that resemble chronic urticaria, but persist for more than 24 h (5). UV may be limited to the skin or present systemically with pulmonary, renal, gastrointestinal, ocular and other types of involvement. Cutaneous manifestations may initially be like urticaria, differing in duration >24 h and usually showing residual post-inflammatory ecchymotic hyperpigmentation (6).

The prevalence and incidence are unclear due to limited data on the disease, variability in disease presentation, and difficulty in classifying it. However, in the United States of America it is reported in 0.5 per 100,000 persons/year, with 12% of cases of leukocytoclastic vasculitis attributed to UV. It is more frequent in women in their fourth decade of life.

The etiology is not identified, however there are some precipitating factors such as: medications, infections (Mononucleosis, Hepatitis B and C, EBV, etc.), autoimmune diseases (Sjögren, SLE), malignancy (lymphomas, leukemias, myelomas, myelodysplasias), complement deficiency and IgG4 deficiency among others (7, 8).

Clinically, it presents as palpable plaques, purpura, ecchymosis, with residual hyperpigmentation, pruritic and sometimes burning or painful (1/3 patients) that last >24 h accompanied by leukocytoclastic vasculitis, in courses of 4 to 8 weeks and resolution in 1 year in 30% of cases.

It is thought that the pathogenesis is provoked by the deposit of immune complexes in the wall of blood vessels, activating the classical complement pathway C3a and C5a, causing degranulation of mast cells and the development of urticarial plaques, also causing increased vascular permeability and chemotaxis of inflammatory cells (neutrophils). C1 is the fundamental activator of the classical complement Pathway. C1q is a collagen-like protein capable of binding to antibodies in immune complexes. Anti C1q antibodies trigger the complement cascade by targeting the collagenous “tail” portion of C1q. Anti-C1q autoantibodies have an important role in HUV (5). HUV/anti-C1q vasculitis is often associated with skin lesion like urticaria, low complement levels and anti C1q antibodies associated with autoimmune diseases like Systemic Lupus Erythematosus, Rheumatoid Arthritis, Proliferative glomerulonephritis, and Chronic obstructive airway disease with worst prognosis than NUV. There is a HUV Syndrome previously known as Mc Duffie Syndrome that refers to a severe chronic idiopathic HUV that involves multiorgan inflammation not associated with another autoimmune disease (8).

UV rays are divided into two groups based on complement levels. (6):

(a) normocomplementemic urticarial vasculitis (NUV). - 80% of cases
(b) hypocomplementemic urticarial vasculitis (HUV) associated with severe systemic disease, associated with autoimmunity, infections and malignancy.

The gold standard for diagnosis is biopsy, finding leukocytoclastic vasculitis, fibrin deposits, perivascular infiltrate of neutrophils, extravasation of erythrocytes and damage to endothelial cells in the biopsy (7).

Antihistamines, oral corticosteroids and omalizumab are the most commonly used treatments for UV. However, antihistamines and corticosteroids have proven to be of little use in UV for their lack of efficacy in former and the latter due to long-term adverse effects. Omalizumab, on the other hand, has shown improvement in 40% of patients with UV (6).

### Erythema multiforme (EM)

Erythema multiforme (EM) is an acute inflammatory disease of the skin and mucous membranes, usually benign, short-term, self-limiting and with a tendency to recur. It is characterized by erythematous raised lesions, with central flattening (target) and blister formation. Its distribution is usually symmetrical, surrounded by a perilesional halo that may resemble an urticarial lesion. The papules may increase in size and adopt the target shape characteristic of erythema multiforme, and epidermal necrosis may develop in the central region of the lesion. EM may present as an isolated skin lesion or with mucocutaneous involvement (9).

They usually appear after an infectious condition such as herpes simplex (most common), streptococcus, coxsackie or even after taking medications (eg: allopurinol, phenobarbital, phenytoin, sulfonamides, ASA, penicillin, erythromycin, and some anti TNF-a (10). Histologically, epidermal necrosis with subdermal blister formation and lymphohistiocytic inflammatory infiltrate is observed. There is no specific treatment for EM, however, in extensive cases, the use of antivirals such as acyclovir is recommended.

### Urticaria pigmentosa (UP)

Urticaria pigmentosa (UP) is part of the dermatoses known as mastocytosis. Mastocytosis is a disorder characterized clonal and pathological accumulation of mast cells by the accumulation of mast cells in the skin, bone marrow, gastrointestinal (GI) tract, liver, spleen, and lymphatic tissues. Cutaneous mastocytosis has 3 presentations depending on the number and type of lesions: (A) Solitary or few lesions (≤3) called “mast cell tumors.” (B) Urticaria pigmentosa (UP), involves multiple lesions ranging from >10 to <100 lesions. (C) Diffuse skin involvement.

UP is the most common benign cutaneous mastocytosis in pediatrics and in general is self-limited by adolescence, although it can occur in adults. Clinically, we detect small brown itchy spots, and when scratched they become edematous, causing a wheal (Darier's sign). Only occasionally it causes complications related to the activation of mast cells in some organ or tissue (11).

### Papular urticaria (PU)

Papular urticaria (PU) is a chronic Th2 cell-mediated hypersensitivity reaction induced by insect bites, which is common in the tropics. It is frequently caused by Aedes aegypti and Culex whoremonger fasciatus mosquitoes, although it may be associated with bed bugs and fleas either by direct reaction or by cross-reaction as in the case of hypersensitivity to cat bugs (Cte f 29 antigen). The role of immunoglobulin E is unclear in this disease. Immunological characterization of the molecular components causing this condition may resolve questions about its pathogenesis (12).

However, there appears to be a relationship with allergic diseases since many children with papular urticaria are atopic, so PU is often sought not only as an isolated pathology but as a comorbid atopic disease (13). The reaction seen in allergic patients with lesions caused by scabies increases the risk of sensitization to mites due to inflammation of the skin with alteration of the skin barrier that enhances the immune response.

### Erythema Marginatum (EM)

Erythema Marginatum (EM) It is a reactive inflammatory erythema, specific to acute rheumatic fever (ARF) that in less than 30% of cases has a history of pharyngeal pain. It presents as a circular, evanescent, non-pruritic erythematous rash with serpiginous edges that can resolve spontaneously and reappear, generally observed on the trunk and extremities. Histologically, there is an infiltration of mononuclear cells with neutrophils in the papillary half and upper part of the reticular dermis. The diagnosis is clinical according to the Jones criteria. It can also be observed in hereditary angioedema and psittacosis (14). This condition is mainly managed with antibiotics from the beta-lactam group such as penicillin, amoxicillin, cephalosporins or macrolides. Recurrence of rheumatic fever should be prevented, since ARF affects the heart, joints, central nervous system and subcutaneous tissue caused by group A streptococcus (14, 15).

### Autoinflammatory syndrome

These are a group of diseases characterized by spontaneous, recurrent or persistent episodes of multisystem inflammation, fever and urticaria. They are caused by alterations in innate immunity, which causes a disregulation of the immune system with abnormal inflammatory activity. IL-1α, IL-1β and IL-1RA are highly regulated inflammatory mediators that intervene in the molecular pattern associated with damage and the pathogen (DAMP and PAMP) and in the cell death pathways in these diseases. Auto-inflammatory syndromes are rare diseases and usually require careful clinical diagnosis since they present with inflammatory manifestations in different organs, joints and tissues. The number of diseases included has been increasing due to advances in genetics and immunology. Examples of autoinflammatory diseases include: Behcet's disease, familial Mediterranean fever (FMF), cryopyrin-associated periodic syndromes (CAPS), TNF receptor-associated periodic syndrome (TRAPS), IL-1 receptor antagonist deficiency (DIRA), hyper IgD syndrome (HIDS), adult-onset Still's disease, VEXAS syndrome, among others.

Treatment may involve the use of biologic agents such as IL-1 inhibitors (anakinra or canakinumab) or TNF-α inhibitors (etanercept), and other therapies (16).

### Urticarial dermatitis (UD)

It is a rarely used term, because UD appears to be a useful histological and clinical term for a subset of dermal hypersensitivity reactions. It is not limited to a specific entity; eczema and drug reactions appear to be the most frequent clinical associations (16). Also known as urticarial dermatitis or eczema, it is characterized by the presence of urticaria-like lesions, but with a more persistent and eczematous component. It is often described as a combination of features of both urticaria and dermatitis. It is an intensely pruritic and recalcitrant skin eruption characterized by erythematous papules and plaques that resemble urticaria but last for more than 24 h and are sometimes accompanied by eczematous lesions (17, 18).

Protein contact dermatitis are cutaneous hypersensitivity reactions following chronic and recurrent exposure or chronic irritation to animal or plant proteins. The mechanisms involved are a mixed combination I/IV (19). This group of conditions includes occupational contact dermatitis, which is a common disease that is almost always irritating (20).

### Bullous pemphigus

Pemphigus encompasses a spectrum of rare mucocutaneous blistering diseases that are autoimmune in origin. Diagnosis is confirmed by biopsy of a small fresh vesicle where IgG and/or C3 deposits are reported on the surface of epidermal keratinocytes. The smooth reticular staining pattern is also known as chicken wire, honeycomb, or net. This is another finding on the direct immunofluorescence test. IgA deposits with an epithelial cell surface pattern in addition to IgG may be present in a subset of cases. Immunoserologic tests such as detection of anti-desmoglein 1 (mucocutaneous PF/PV) and/or anti-desmoglein 3 IgG (mucosal or mucocutaneous PV) autoantibodies by ELISA (mannose-binding lectin) are usually found (21).

The infantile presentation is usually severe, with blisters on the hands and feet present in all cases. The pathogenesis and diagnostic criteria are comparable to those of PA in adults, however, BP180 NC16A ELISA levels appear to be significantly higher in infants. The overall prognosis of the disease is favorable (22, 23).

## Conclusion

Urticaria is a condition with edema of the dermis and occasionally with edema of the subcutaneous cellular tissue, which is pruritic and transient. However, all pruritic conditions present with redness and itching, which makes them initially confused with urticaria. This article attempts to clarify some differences to achieve a better diagnosis.
